# APE1-mediated DNA damage repair provides survival advantage for esophageal adenocarcinoma cells in response to acidic bile salts

**DOI:** 10.18632/oncotarget.7696

**Published:** 2016-02-25

**Authors:** Jun Hong, Zheng Chen, Dunfa Peng, Alexander Zaika, Frank Revetta, M. Kay Washington, Abbes Belkhiri, Wael El-Rifai

**Affiliations:** ^1^ Department of Surgery, Vanderbilt University Medical Center, Nashville, Tennessee, USA; ^2^ Department of Cancer Biology, Vanderbilt University Medical Center, Nashville, Tennessee, USA; ^3^ Department of Veterans Affairs, Tennessee Valley Healthcare System, Nashville, Tennessee, USA; ^4^ Department of Pathology, Vanderbilt University Medical Center, Nashville, Tennessee, USA

**Keywords:** APE1, acidic bile salts, JNK, p38, base excision repair

## Abstract

Chronic Gastroesophageal Reflux Disease (GERD) is the main risk factor for the development of Barrett's esophagus (BE) and its progression to esophageal adenocarcinoma (EAC). Accordingly, EAC cells are subjected to high levels of oxidative stress and subsequent DNA damage. In this study, we investigated the expression and role of Apurinic/apyrimidinic endonuclease 1 (APE1) protein in promoting cancer cell survival by counteracting the lethal effects of acidic bile salts (ABS)-induced DNA damage. Immunohistochemistry analysis of human tissue samples demonstrated overexpression of APE1 in more than half of EACs (70 of 130), as compared to normal esophagus and non-dysplastic BE samples (*P* < 0.01). To mimic *in vivo* conditions, we treated *in vitro* cell models with a cocktail of ABS. The knockdown of endogenous APE1 in EAC FLO-1 cells significantly increased oxidative DNA damage (*P* < 0.01) and DNA single- and double-strand breaks (*P* < 0.01), whereas overexpression of APE1 in EAC OE33 cells reversed these effects. Annexin V/PI staining indicated that the APE1 expression in OE33 cells protects against ABS-induced apoptosis. In contrast, knockdown of endogenous APE1 in FLO-1 cells increased apoptosis under the same conditions. Mechanistic investigations indicated that the pro-survival function of APE1 was associated with the regulation of stress response c-Jun N-terminal protein kinase (JNK) and p38 kinases. Pharmacological inhibition of APE1 base excision repair (BER) function decreased cell survival and enhanced activation of JNK and p38 kinases by ABS. Our findings suggest that constitutive overexpression of APE1 in EAC may be an adaptive pro-survival mechanism that protects against the genotoxic lethal effects of bile reflux episodes.

## INTRODUCTION

The incidence of esophageal adenocarcinoma (EAC) has risen rapidly over the past few decades in the United States and the Western world [1, 2]. Barrett's esophagus (BE) develops as a consequence of chronic gastroesophageal reflux disease (GERD), where gastric juice containing acid and bile salts abnormally refluxate into the distal region of esophagus [3–5]. BE is a premalignant metaplastic columnar epithelial lesion that can progress to EAC through intermediate stages of low- and high-grade dysplasia (reviewed by [6]). This abnormal exposure of esophageal cells to a mixture of acid and bile salts is not limited to BE, but exists in the continuum of neoplastic progression to EAC. This unique etiology makes EAC an excellent example of reactive oxygen species (ROS)-induced cancer.

Previous studies have shown that short exposure to bile salts and low pH induces oxidative stress and DNA damage in esophageal tissues and cells [7, 8]. In fact, acidic bile salts treatment increases the levels of ROS, leading to an increase in the levels of 8-hydroxy-deoxyguanosine (8-OH-dG), a marker of oxidative DNA damage, and p-H2AX (S139), a marker of DNA double-strand breaks, in esophageal cells [9, 10]. Oxidative stress induces multiple signal transduction pathways, among which are the c-Jun N-terminal protein kinases (JNK) and p38 pathways. These stress response mitogen-activated protein kinases (MAPKs) regulate a variety of cellular activities, including cell growth, differentiation, survival, and death [11, 12]. Studies have shown that acidic bile salts, as well as other genotoxic agents, induce oxidative stress and activation of JNK and p38 pathways; thereby, modulating the cell apoptotic response in human cells [13–16]. Previous reports have shown that several genes that function as ROS-buffering system in normal cells are silenced in EAC [17–19], raising important questions about regulation of oxidative DNA damage levels in cancer cells. This chronic exposure to high levels of ROS in neoplastic Barrett's generates high levels of oxidative DNA damage, if unrepaired will lead to accumulation of lethal levels of DNA damage.

Apurinic/apyrimidinic endonuclease 1 (APE1) (also called Ref-1, HAP1, or APEX) is one of the key enzymes of the base excision repair (BER) pathways that are critical in repairing oxidative DNA damage in mammals [20]. Apurinic/apyrimidinic (AP) sites are one of the major types of oxidative DNA damage generated by ROS [21, 22] and are repaired primarily by BER pathways [23]. The basic reactions of BER pathways have been extensively reviewed by [20, 23, 24]. APE1 is also a reductive activator of different transcription factors such as nuclear factor-κB (NF-κB), p53, hypoxia inducible factor-1α (HIF-1α), cAMP response element binding protein (CREB), and activator protein-1 (AP-1) [25].

The primary aim of this study was to investigate the role of APE1 in survival of EAC cells in response to acidic bile salts, and identify the underlying molecular mechanism. We have uncovered that APE1 protein is expressed at high levels in human EAC tissue samples. We also demonstrated that APE1 promotes the survival of EAC cells in response to acidic bile salts through enhancing DNA damage repair and attenuating stress response JNK- and p38-mediated apoptosis. These findings suggest that APE1 might provide a survival advantage for EAC cells in response to acidic bile salts-induced oxidative stress.

## RESULTS

### High expression levels of APE1 are associated with diminished acidic bile salts-induced DNA damage in esophageal adenocarcinomas

**Figure 1:**
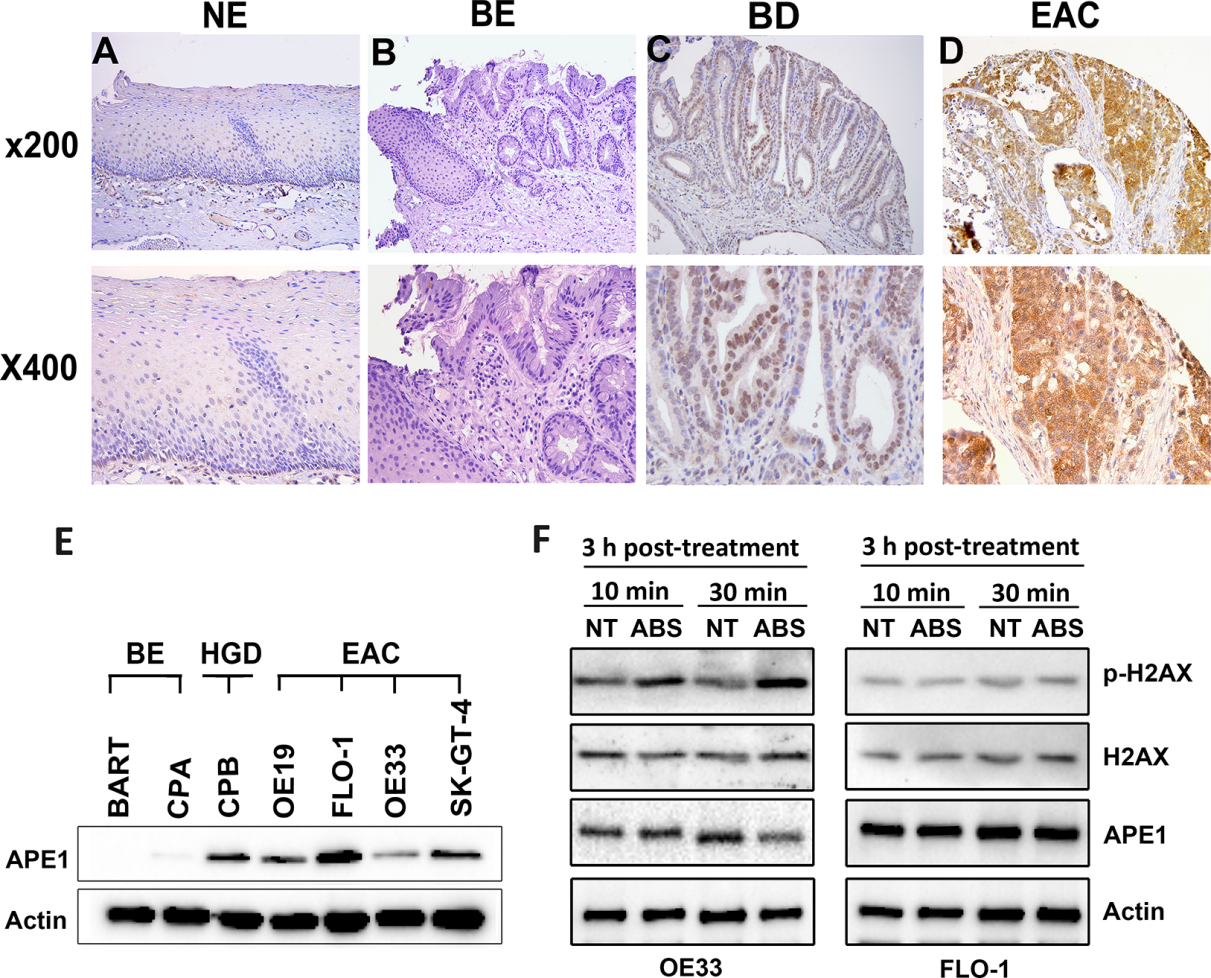
APE1 is overexpressed in esophageal adenocarcinomas and associated with decreased acidic bile salts-induced DNA damage (**A**–**D**) A representative APE1 IHC staining of normal esophagus (NE, A), non-dysplastic Barrett's esophagus (BE, B), dysplastic Barrett's esophagus (BD, C), and esophageal adenocarcinoma (EAC, D). As shown, weak to absent immunostaining was observed in normal and BE tissues (A and B), whereas moderate nuclear staining with weak-moderate cytosolic staining was observed in dysplastic BE (C). EAC samples demonstrate strong nuclear and cytosolic immunostaining (D). (**E**) Western blot analysis of APE1 is shown in a panel of non-dysplastic BE (BE), high-grade dysplasia (HGD), and EAC cells. (**F**) Western blot analysis is shown for p-H2AX (S139), H2AX, and APE1 proteins in OE33 and FLO-1 cells non-treated or treated with acidic bile salts.

We investigated the expression of APE1 in normal tissues (stomach, esophagus, kidney and liver), non-dysplastic BE, dysplastic BE, and EAC tissue samples by Immunohistochemistry (IHC). APE1 IHC showed absent to weak nuclear immunostaining in all normal tissues (Supplementary Figure S1) and non-dysplastic BE (Figure 1). Barrett's esophagus with dysplasia demonstrated moderate immunostaining that was predominantly nuclear with a diffuse cytosolic pattern. In contrast, more than half of EACs tissue samples demonstrated significantly higher expression levels of APE1 (*P* < 0.01) than normal and non-dysplastic BE tissues, showing aberrant moderate to strong (CES range from 4 to 12) nuclear and cytosolic immunostaining (Figure 1D). A summary of IHC scores is given in Supplementary Table S1. We next evaluated the APE1 protein expression by Western blot analysis in a panel of Barrett's cell models; non-dysplastic Barrett's (BE), high-grade dysplastic (HGD) and EAC cell lines. Consistent with the expression pattern in human tissues, we detected high expression level of APE1 in dysplastic BE and EAC cell lines (Figure 1E). Among the EAC cell lines, FLO-1 exhibited the highest and OE33 the lowest endogenous levels of APE1 expression (Figure 1E). Neoplastic Barrett's cells (HGD and EAC) are exposed to high levels of oxidative stress due to activation of oncogenic pathways and chronic exposure to bile reflux. Because of the high expression levels of APE1 in neoplastic Barrett's (HGD and EAC) and its role in DNA repair, we evaluated the DNA damage levels by Western blot analysis of p-H2AX (S139) in response to acidic bile salts in OE33 and FLO-1 EAC cell lines with different levels of APE1 expression. We treated the cells with acidic bile salts cocktail (200 μM, pH 4) for 10 min or 30 min followed by incubation in complete media for 3 h post-treatment. We found that p-H2AX was substantially induced in response to acidic bile salts in OE33 cells, which exhibit low APE1 expression (Figure 1F). However, in FLO-1 cells expressing a high level of APE1, there was no noticeable induction of p-H2AX by acidic bile salts (Figure 1F). These results suggest a negative correlation between APE1 expression and acidic bile salts-induced DNA damage levels in EAC.

### APE1 suppresses acidic bile salts-induced DNA damage and apoptosis

**Figure 2:**
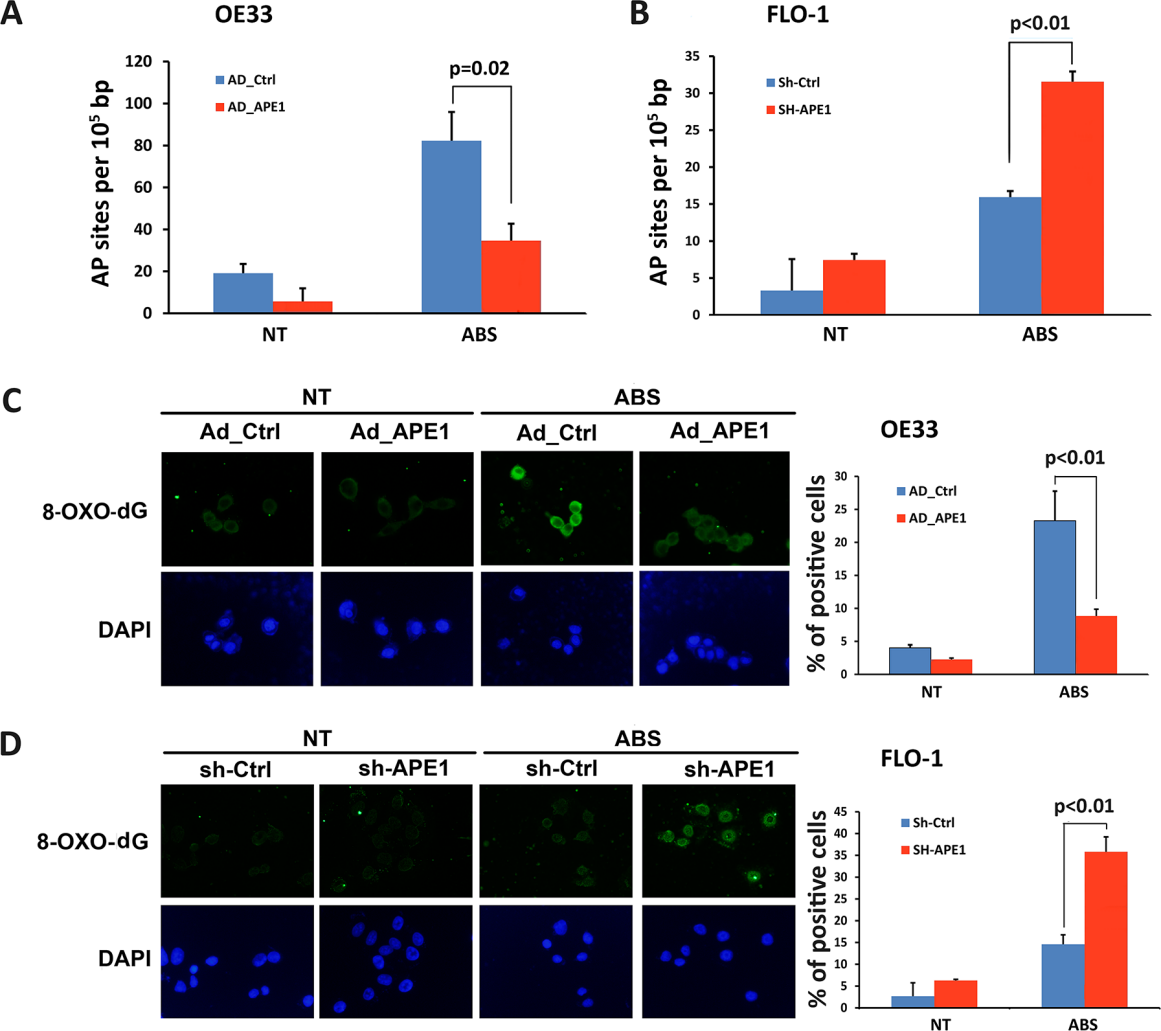
APE1 decreases acidic bile salts-induced oxidative DNA damage in EAC cells Cells were non-treated or treated with acidic bile salts (200 μM, pH4.0) for 30 min, followed by recovery in complete media for 3 h, and subjected to relevant assays to measure AP sites (OxiSelect^™^; Cell BioLabs, San Diego, CA, USA) and immunofluorescence staining with an antibody against 8-OH-dG as described in Materials and Methods. AP sites in control adenovirus or APE1 adenovirus infected OE33 cells (**A**) and in control shRNA or APE1 shRNA stably transfected FLO-1 cells (**B**) in response to acidic bile salts. Immunofluorescence for 8-OH-dG in control adenovirus or APE1 adenovirus infected OE33 cells (**C**) and in control shRNA or APE1 shRNA stably transfected FLO-1 cells (**D**) following treatment with acidic bile salts. Quantification analyses are shown on the right panels (C and D) The results are representative of at least three independent experiments.

To investigate the function of APE1 in regulating acidic bile salts-induced DNA damage and cancer cell survival, we used OE33 and FLO-1 EAC cell lines with low and high levels of APE1, respectively. We investigated whether modulations of APE1 expression level affect apurinic/apyrimidinic (AP) sites accumulation in response to acidic bile salts. We treated OE33 cells, following overexpression of APE1, and FLO-1 cells, after APE1 knockdown, with acidic bile salts for 30 min followed by incubation in regular complete media for 3 h post-treatment, and then measured AP sites. We found that the expression of APE1 significantly attenuated AP sites accumulation in response to acidic bile salts in OE33 cells (*P* = 0.02, Figure 2A). The knockdown of endogenous APE1 in FLO-1 cells significantly increased acidic bile salts-induced accumulation of AP sites (*P* < 0.01, Figure 2B). We next examined levels of oxidative DNA damage induced by acidic bile salts following modulations of APE1 expression. The data indicated that the expression of APE1 in OE33 cells significantly reduced oxidative DNA damage, as indicated by a decreased 8-OH-dG level, in response to acidic bile salts as compared to control cells (*P* < 0.01, Figure 2C). In contrast, knockdown of endogenous APE1 in FLO-1 cells significantly enhanced acidic bile salts-induced oxidative DNA damage as depicted by an increased 8-OH-dG level relative to control cells (*P* < 0.01, Figure 2D).

**Figure 3:**
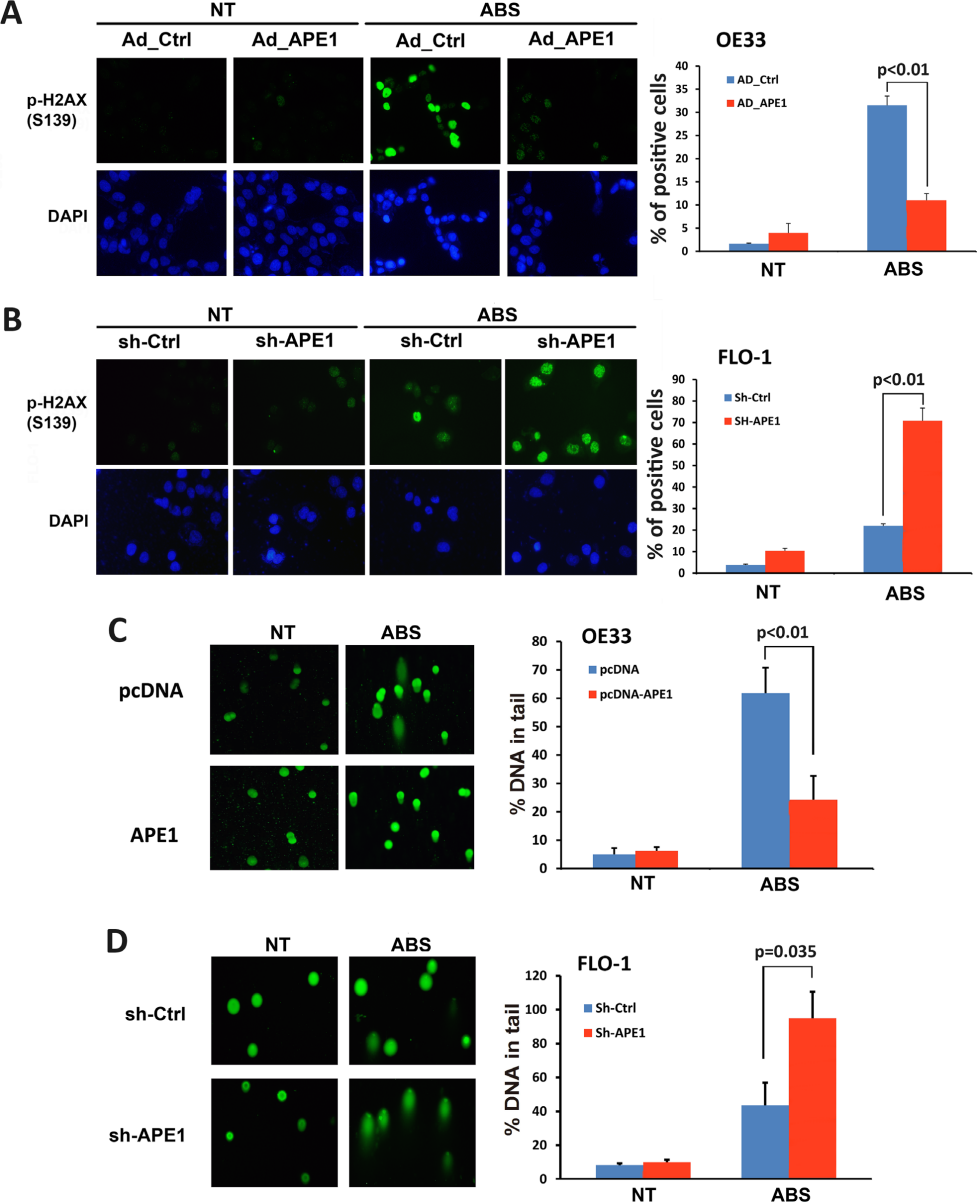
APE1 decreases acidic bile salts-induced double strand DNA damage in EAC cells Cells were non-treated or treated with acidic bile salts (200 μM, pH 4.0) for 30 min followed by recovery in complete media for 3 h and subjected to immunofluorescence and comet assays. Immunofluorescence for p-H2AX (S139) in control adenovirus or APE1 adenovirus infected OE33 cells (**A**) and in control shRNA or APE1 shRNA stably transfected FLO-1 cells (**B**). Quantification analyses of immunofluorescence data are shown on the right panels A and B. Comet assay in OE33 cells stably expressing empty vector (pcDNA) or APE-1 (**C**) and FLO-1 cells stably expressing control shRNA or APE-1 shRNA (**D**). Quantification analyses are shown on the right panels C and D. The results are representative of at least three independent experiments.

If left unrepaired, oxidative DNA damage as an early molecular event can progress to DNA double-strand breaks. Therefore, we investigated the role of APE1 in regulating acidic bile salts-induced double-strand DNA damage. We performed immunofluorescence for p-H2AX (S139), a marker of double-strand DNA damage. Expression of APE1 in OE33 cells significantly decreased acidic bile salts-induced double-strand DNA damage relative to control cells as indicated by reduced p-H2AX (S139) level (*P* < 0.01, Figure 3A). Conversely, knockdown of endogenous APE1 in FLO-1 cells significantly enhanced acidic bile salts-induced double-strand DNA damage relative to control cells as shown by an increased p-H2AX (S139) level (*P* < 0.01, Figure 3B). We further confirmed the role of APE1 in regulating DNA damage using comet assay, which detects both DNA single- and double-strand breaks. Our data indicated that the expression of APE1 in OE33 cells significantly reduced acidic bile salts-induced DNA damage relative to control cells (*P* < 0.01, Figure 3C). However, knockdown of endogenous APE1 in FLO-1 cells significantly increased acidic bile salts-induced DNA damage as compared to control cells (*P* = 0.035, Figure 3D). Taken together, these data indicated that APE1 expression in EAC cells protects against double-strand DNA damage in response to acidic bile salts.

**Figure 4:**
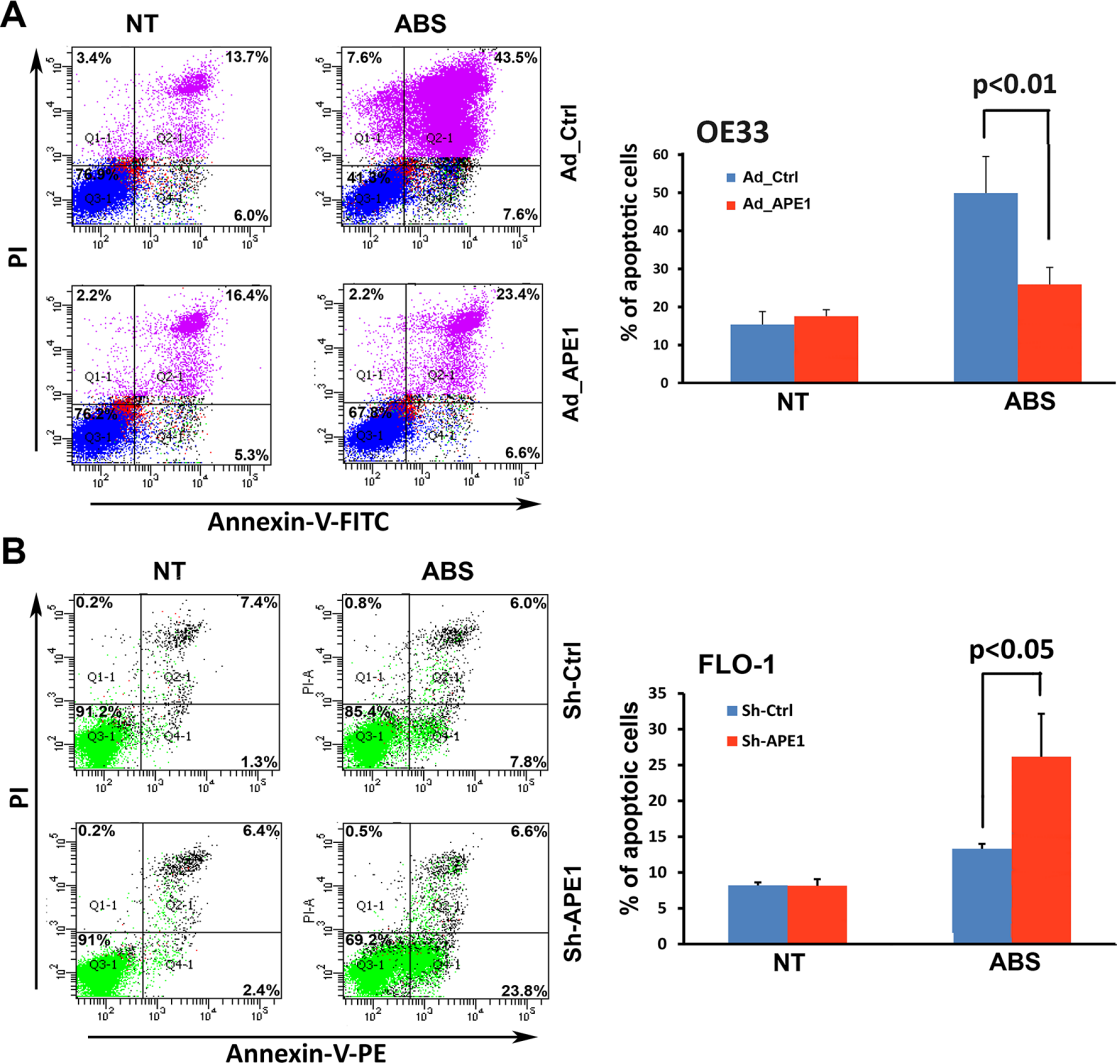
APE1 attenuates apoptosis in response to acidic bile salts in EAC cells Cells were non-treated or treated with acidic bile salts (200 μM, pH 4.0) for 30 min followed by recovery in complete media for 3 h, and subjected to Annexin V/PI staining. Annexin V/PI staining of control adenovirus or APE1 adenovirus infected OE33 cells (**A**) and FLO-1 cells stably expressing control shRNA or APE1 shRNA (**B**). The results are representative of three independent experiments.

Unrepaired oxidative DNA damage lesions could progress to DNA single- and double-strand breaks, leading to apoptotic cell death [26, 27]. As our data clearly showed that APE1 decreases acidic bile salts-induced DNA damage in EAC cells, we investigated whether APE1 could promote cell survival. We evaluated apoptosis following APE1 expression and knockdown using Annexin-V/PI staining following treatment with acidic bile salts. The data indicated that treatment with acidic bile salts induced 51.1% and 30% apoptotic cell death in control OE33 cells and APE1 expressing OE33 cells, respectively (*P* < 0.01, Figure 4A). Treatment with acidic bile salts induced 30.4% apoptotic cell death in APE1 knockdown FLO-1 cells as opposed to 13.8% apoptotic cell death in control FLO-1 cells (*P* < 0.05, Figure 4B). Together, these results indicated that APE1 plays a pivotal role in protecting against DNA damage and cell death, in response to genotoxic stimuli such as acidic bile salts exposure, a common pathological condition in reflux-induced Barrett's carcinogenesis and EAC.

### APE1 reduces DNA damage-induced apoptosis through regulation of JNK and p38 signaling

**Figure 5:**
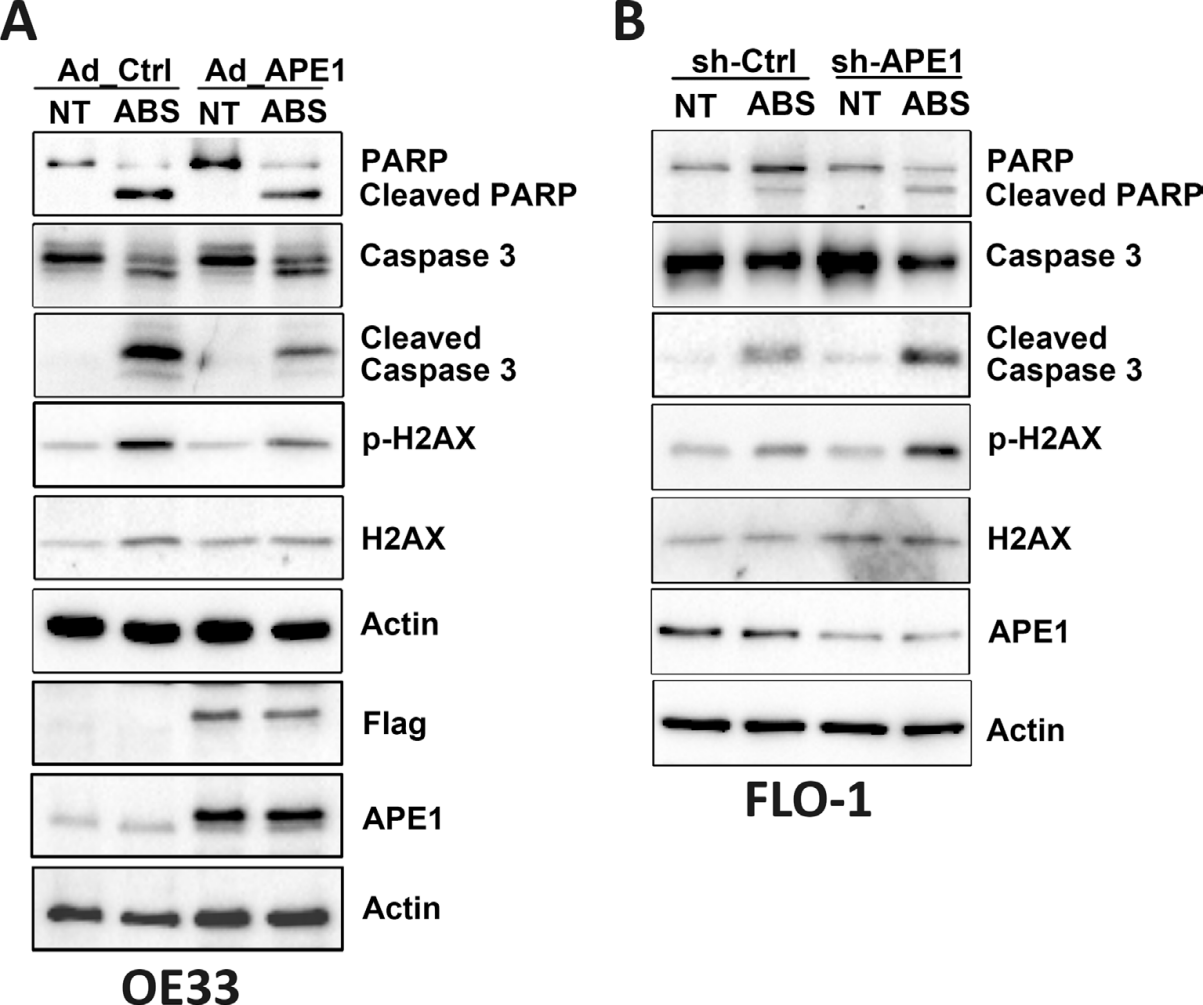
APE1 regulates acidic bile salts-induced DNA damage and apoptosis signaling Cells were non-treated or treated with acidic bile salts (200 μM, pH 4.0) for 30 min followed by recovery in complete media for 3 h, and subjected to Western blot analysis of the indicated proteins. β-actin was used as a loading control. OE33 cells (**A**) were infected with control or APE1 adenoviruses. FLO-1 cells (**B**) were stably transfected with control shRNA or APE1 shRNA.

We have shown that acidic bile salts-mediated DNA damage can lead to apoptotic cell death, and demonstrated that APE1 counteracts these effects in EAC cells. Therefore, we investigated the mechanistic role of APE1 in regulating stress response-mediated apoptosis. We used APE1 overexpression and knockdown in esophageal cell models. Western blot analysis data indicated that the expression of APE1 significantly decreased acidic bile salts-induced p-H2AX (S139) protein levels and cleavage of caspase-3 and PARP in OE33 cells (Figure 5A). Similar results were observed in CPB cells (Supplementary Figure S2A). In contrast, knocking down endogenous APE1 substantially increased acidic bile salts-induced p-H2AX (S139) protein level and cleavage of caspase-3 and PARP in FLO-1 cells (Figure 5B).

**Figure 6:**
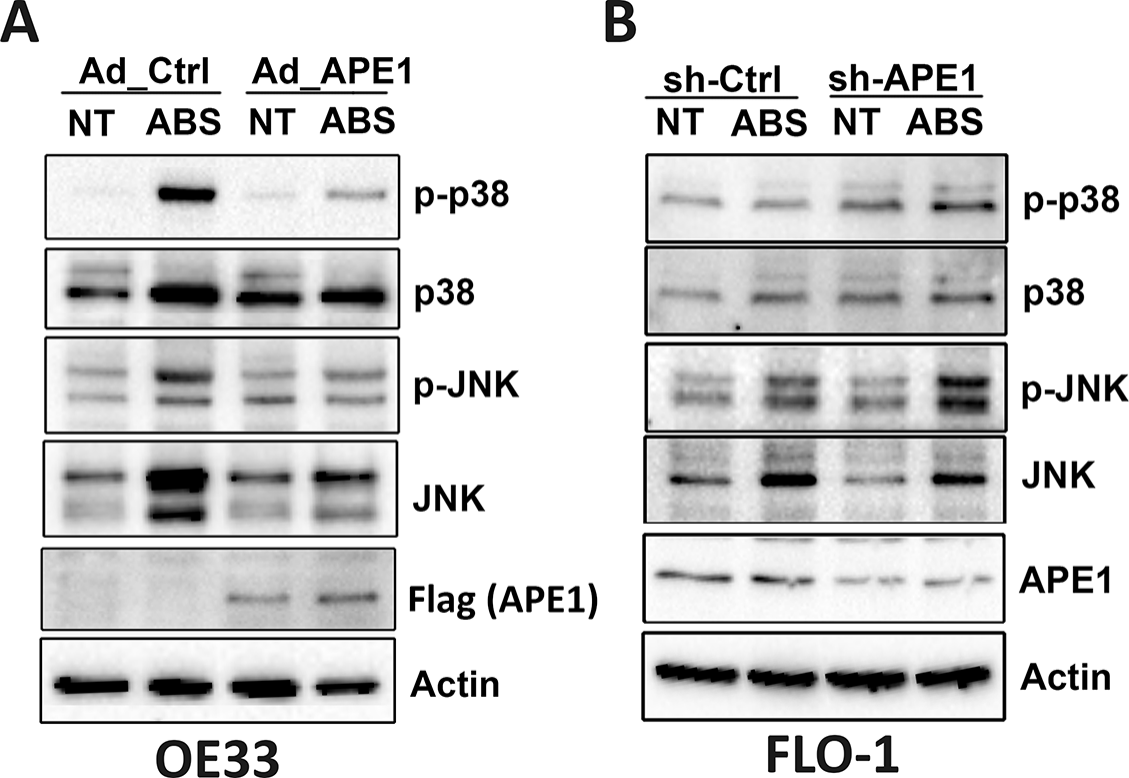
APE1 regulates acidic bile salts-induced activation of JNK and p38 pathways Cells were non-treated or treated with acidic bile salts (200 μM, pH 4.0) for 30 min followed by recovery in complete media for 3 h, and subjected to Western blot analysis of the indicated proteins. β-actin was used as a loading control. OE33 cells (**A**) were infected with control or APE1 adenoviruses. FLO-1 cells (**B**) were stably transfected with control shRNA or APE1 shRNA.

Previous studies have shown that DNA damage induces cell death through activation of stress response JNK and p38 MAPK pathways [13, 16]. This is especially evident in p53-mutant EAC cells as shown in our mutant cell models (OE33, FLO-1 and CPB) (Figure 6 and Supplementary Figures S2 and S3). In fact, we observed activation of JNK and p38 kinases that was associated with increased cell death in response to acidic bile salts. These effects were significantly suppressed following pharmacological inhibition of JNK and p38, suggesting that stress response JNK and p38 kinases are required to mediate apoptosis in EAC cell models (Supplementary Figure S4). Therefore, we investigated whether APE1 regulates the activation of JNK and p38 in response to acidic bile salts in APE1 overexpression and knockdown esophageal cell models. We found that the expression of APE1 in OE33 cells decreased acidic bile salts-induced JNK, p-JNK (T183/Y185), p38, and p-p38 (T180/Y182) protein levels as compared to control cells (Figure 6A). We found similar results under the same experimental conditions in CPB cells (Supplementary Figure S2B). Conversely, knockdown of endogenous APE1 in FLO-1 cells substantially increased acidic bile salts-induced p-JNK (T183/Y185) and p-p38 (T180/Y182) protein levels as compared to control cells (Figure 6B). These results strongly suggested that APE1 suppression of acidic bile salts-induced DNA damage reduces JNK- and p38-mediated apoptosis.

### APE1 base excision repair activity is required for cell survival in response to acidic bile salts

**Figure 7:**
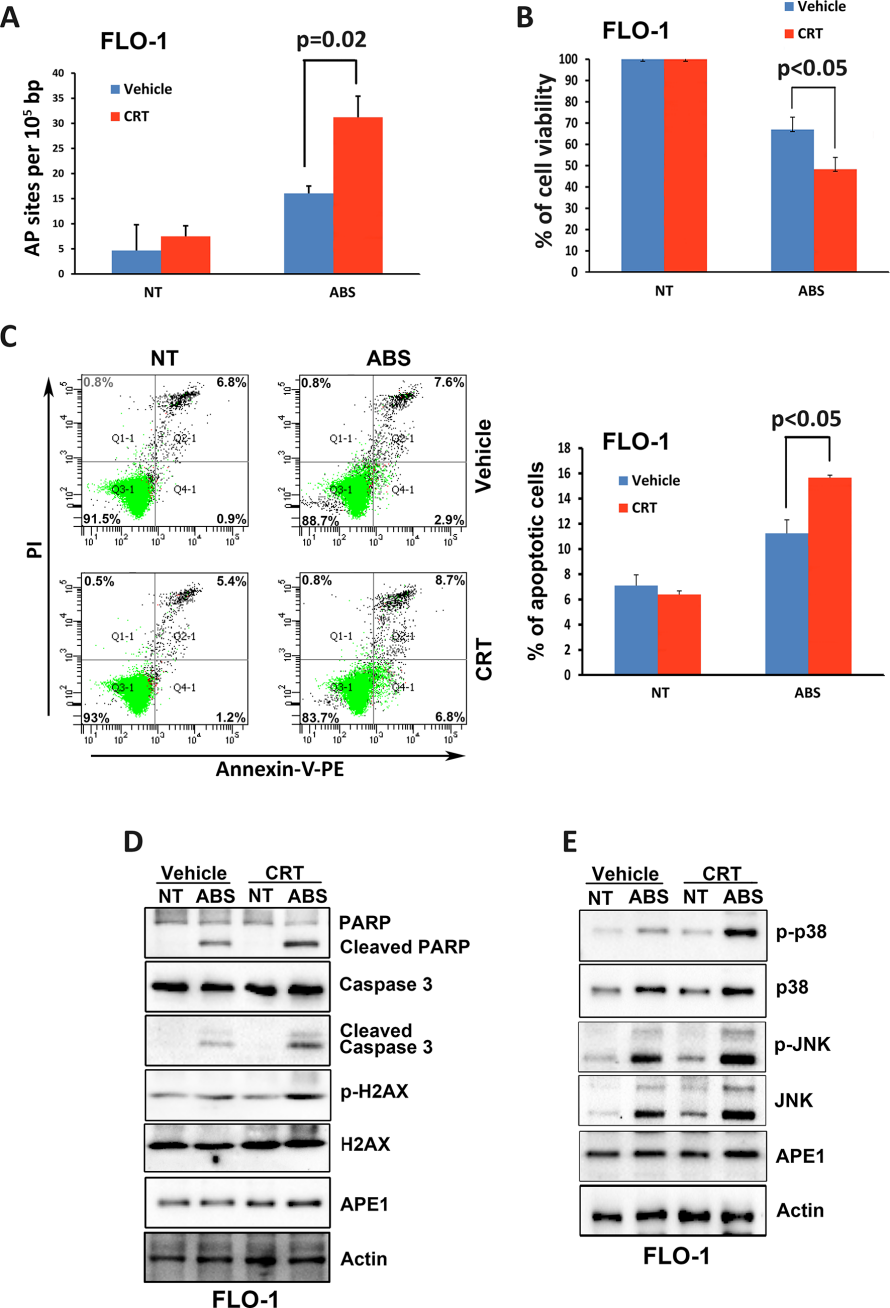
Inhibition of APE1 DNA repair activity enhances DNA damage, apoptosis and activation of JNK and p38 pathways in response to acidic bile salts FLO-1 cells were non-treated or treated with acidic bile salts (200 μM, pH 4.0) alone or in combination with CRT (100 μM) for 30 min, followed by recovery in complete media for 3 h and subjected to AP sites and CellTiter-Glo assays, Annexin V/PI staining, and Western blotting. AP sites (**A**), CellTiter-Glo (**B**), Annexin V/PI (**C**), and Western blot analysis (**D** and **E**) data are shown. β-actin was used as a loading control. Quantification analysis of Annexin V/PI staining data is shown on the right panel C.

To investigate whether the base excision repair function of APE1 is critical for suppressing acidic bile salts-induced apoptosis, we used a specific APE1 pharmacological inhibitor, 7-Nitroindole-2-carboxylic acid (CRT). Our data indicated that inhibition of APE1 with CRT (100 μM for 30 min) significantly increases the AP sites accumulation in response to treatment with acidic bile salts (200 μM for 30 min) (*P* = 0.02, Figure 7A). Accordingly, the CellTiter-Glo assay data indicated that inhibition of APE1 with CRT sensitized FLO-1 cells to acidic bile salts as indicated by significantly decreased cell survival (*P* < 0.05, Figure 7B). In addition, the Annexin-V/PI data showed that inhibition of APE1 with CRT in combination with acidic bile salts in FLO-1 cells significantly increased apoptosis by 47.6% relative to treatment with acidic bile salts alone (*P* < 0.05, Figure 7C). The inhibition of APE1 with CRT in combination with acidic bile salts treatment in FLO-1 cells increased protein levels of p-H2AX (S139), cleaved PARP, and cleaved caspase-3 as compared to cells treated with acidic bile salts alone (Figure 7D). Moreover, there was a significant increase of the p-JNK (T183/Y185) and p-p38 (T180/Y182) protein levels in the combination treated cells relative to cells treated with acidic bile salts alone (Figure 7E). These results clearly indicated that APE1 base excision repair activity is required for promoting cell survival in response to acidic bile salts through reducing DNA damage, thereby suppressing activation of stress response JNK and p38 pathways.

## DISCUSSION

Acidic bile salts, which are major components of gastroesophageal refluxate, have been implicated in the development of BE and EAC [28, 29]. In fact, acidic bile salts induce oxidative stress and DNA damage, leading to apoptosis in human normal esophageal mucosal epithelial cells [10, 30–32]. Several antioxidant enzymes that suppress ROS levels in normal cells are silenced in EAC [17–19]. Nevertheless, EAC develops in an acidic bile salts-induced genotoxic stress environment, a unique condition for carcinogenesis. To maintain DNA damage at tolerable and below lethal levels, these cancer cells must acquire adaptive and protective mechanisms presumably through the activation of pro-survival pathways. The objective of this study was to investigate the role of APE1 in survival of EAC cells in response to acidic bile salts, and uncover the underlying molecular mechanism.

Our data showed that APE1 protein is frequently expressed at high levels in dysplastic Barrett's and EAC human tissue samples and cell lines. We found an inverse correlation between APE1 expression and DNA damage induced by acidic bile salts in cell models. These findings strongly suggested a potential functional role for APE1 in EAC. In support of this hypothesis, a report by Wang and colleagues [33] indicated that APE1 protein expression was elevated in osteosarcoma tissues, and there was a significant direct association between high APE1 expression levels and reduced overall survival of patients.

On the basis that APE1 was implicated in DNA damage repair through its BER function in some cancers [24, 34], we evaluated the role of APE1 in the regulation of acidic bile salts-induced DNA damage in EAC cells. We found that modulation of APE1 expression affects oxidative DNA damage, as indicated by AP sites accumulation or 8-OH-dG staining, in response to acidic bile salts in EAC cells. Our data showed that the expression of APE1 significantly attenuated oxidative DNA damage. Conversely, knocking down of APE1 expression significantly increased oxidative DNA damage. It has been shown that unrepaired oxidative DNA damage could progress to DNA single- and double-strand breaks, leading to apoptotic cell death [35]. Notably, acidic bile salts treatment causes DNA double-strand breaks, as indicated by substantial increases in p-H2AX (S139) protein levels, in esophageal cell lines [9, 36]. In response to acidic bile salts treatment, we found that DNA double-strand breaks levels were significantly reduced after expression of APE1, and increased after knocking down of APE1 in EAC cells. Our comet assay data confirmed the role of APE1 in reducing DNA single- and double-strand breaks in response to acidic bile salts in EAC cells. Collectively, these findings demonstrate that APE1 attenuates acidic bile salts-induced DNA damage in EAC cells. Oxidative stress and DNA damage lead to apoptotic cell death through activation of p53 as part of a DNA damage response [37, 38]. Since our data clearly showed that APE1 was involved in DNA damage repair, we postulated that APE1 could promote cell survival in response to acidic bile salts. Indeed, our results indicated that expression of APE1 substantially decreased apoptosis and knocking down of APE1 markedly increased apoptosis in response to acidic bile salts. These findings were supported by the relevant changes in the molecular markers of apoptosis. Previous reports indicated that DNA damage-induced apoptosis involves activation of stress response JNK and p38 MAPKs [13, 16]. In fact, one report showed that RITA, a DNA damaging agent, induces cancer cell death independent of p53 function via activation of JNK and p38 [39–43]. This is particularly relevant to our EAC cell models which express mutant p53 but still undergo apoptotic cell death in response to DNA damaging agents [44, 45]. Reports indicated that the mechanisms of resistance to acidic bile salts include activation of NF-ĸB, the up-regulation of BCL-2 and inactivation of BAX [46, 47]. However, our data demonstrated that APE1 promotes the survival of EAC cells in response to acidic bile salts through suppressing the activation of JNK and p38 pathways. While we have not shown that APE1 directly inhibits JNK and p38 MAPKs, our data strongly suggested that APE1 reduction of acidic bile salts-induced DNA damage attenuates JNK and p38 mediated apoptosis. In addition to its redox function, APE1 is one of the key enzymes of BER pathways, which repairs oxidative DNA damage caused by endogenous and exogenous agents in mammals [20]. Notably, AP sites are one of the major types of oxidative DNA damage [21] and are repaired primarily by BER pathways [23]. Unrepaired AP sites can progress to DNA single- and double-strand breaks, leading to cytotoxicity and apoptosis [35]. CRT, a selective inhibitor of APE1 BER activity, potentiates the cytotoxicity of several DNA base-targeting compounds [48]. One study showed that inhibition of APE1 with CRT at acidic environment induces an increase in p-H2AX level and cleavage of PARP-1 in HCT116 cells [49]. We confirmed that inhibition of APE1 BER activity with CRT significantly increased AP sites accumulation, DNA double-strand breaks, activation of JNK and p38 pathways, and apoptosis in response to acidic bile salts.

**Figure 8:**
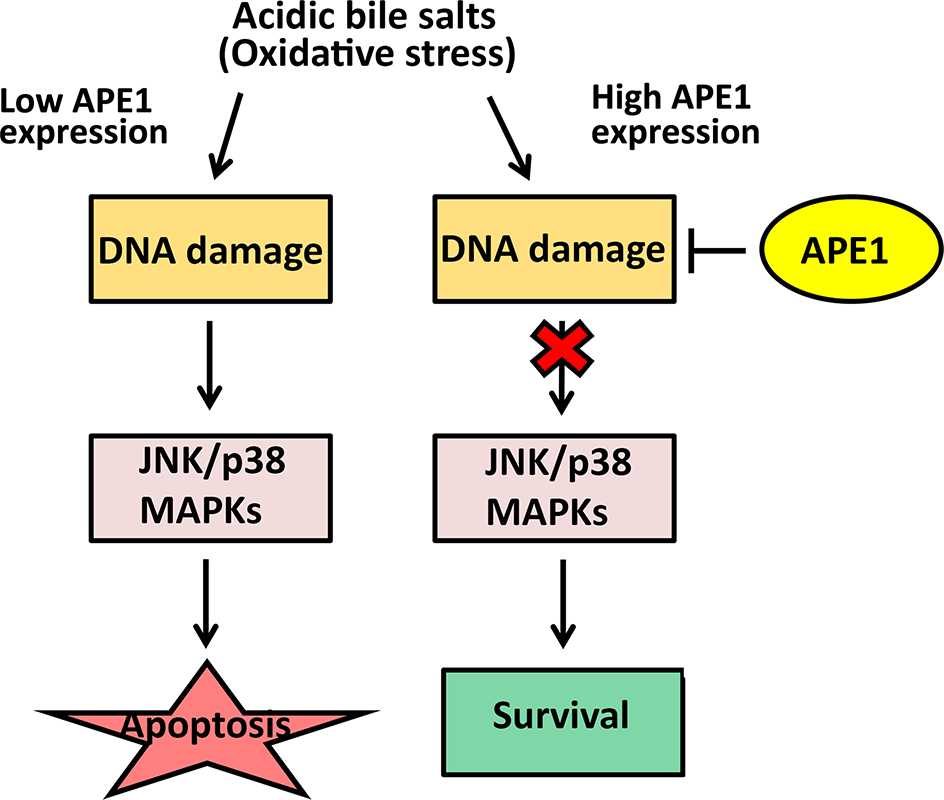
APE1-mediated DNA damage repair promotes cell survival in response to acidic bile salts in mutant p53-expressing EAC cells Under oxidative stress condition induced by acidic bile salts, cells with low expression of APE1 sustain high levels of DNA damage that lead to activation of JNK and p38 MAPKs and subsequent apoptotic cell death. In contrast, under the same condition, cells expressing high levels of APE1sustain less DNA damage because of APE1 BER activity. This results in less activation of JNK and p38 MAPKs, leading to increased cell survival.

In summary, our results indicated that high expression levels of APE1 in EAC underlie an adaptive acidic bile salts resistance phenotype. We showed that APE1 promotes cell survival in response to acidic bile salts through enhancing DNA damage repair. This ultimately suppresses activation of stress response JNK and p38 kinases in EAC (Figure 8). These findings also suggest that APE1 might enhance Barrett's carcinogenesis through promotion of cell survival in condition of acidic bile salts-induced oxidative stress. Notably, recent data using investigational APE1 inhibitors in preclinical models suggest a potential therapeutic benefit of targeting APE1 [50–52]. Our data provide evidence suggesting that APE1 could be exploited as a therapeutic target to sensitize tumors to DNA-damaging drugs in EAC.

## MATERIALS AND METHODS

### Cell lines and reagents

The human esophageal adenocarcinoma cell lines (OE33 and FLO-1) were kindly provided by Dr. David Beer (University of Michigan, Ann Arbor, MI). OE19 and SK-GT4 cells were obtained from Sigma-Aldrich (St. Louis, MO). OE33 and OE19 cells were maintained in RPMI medium (GIBCO, Carlsbad, CA) supplemented with 10% fetal bovine serum (FBS) (Invitrogen Life Technologies, Carlsbad, CA) and 1% penicillin/streptomycin (GIBCO). SK-GT-4 and FLO-1 cells were maintained in DMEM medium (GIBCO) supplemented with 10% FBS and 1% penicillin/streptomycin. The immortalized BART (non-dysplastic BE) was a kind gift from Dr. Rhonda Souza at University of Texas Southwestern whereas CPA (non-dysplastic BE) and CPB (BE with high-grade dysplasia) cells were purchased from ATCC (Manassas, VA); all cells were grown in accordance with supplier conditions. APE1, p-H2AX (S139), H2AX, caspase-3, PARP, and β-actin antibodies were obtained from Cell Signaling Technology (Danvers, MA). p-JNK (T183/Y185), JNK, p-p38 (T180/Y182), and p38 antibodies were obtained from Santa Cruz Biotechnology (Santa Cruz, CA). 8-OH-dG monoclonal antibody (clone 2E2) was purchased from Trevigen Inc., Gaithersburg, MD.

### APE1 expression and plasmids

A full length of APE1 coding sequence with Flag tag was amplified from cDNA by PCR using Platinum PCR SuperMix High Fidelity (Invitrogen) and was cloned into pcDNA3.1. The APE1 coding sequence from pcDNA3.1/APE1 plasmid was sub-cloned into Xba I and BamH I restriction sites of the adenoviral shuttle vector (pACCMV). The recombinant adenovirus expressing APE1 was generated by co-transfecting HEK-293 cells with the shuttle and backbone adenoviral (pJM17) plasmids using the Calcium Phosphate Transfection kit (Applied Biological Materials Inc., Richmond, BC).

### Small hairpin RNA (shRNA)

Lentivirus particles expressing control shRNA or a cocktail of five different clones of APE1 shRNA were produced and validated by Sigma-Aldrich and then utilized to transduce cells. FLO-1 cells that express high levels of endogenous APE1 were transduced with lentiviruses expressing control shRNA or APE1 shRNA following standard protocols. Stably transduced cells were selected by addition of puromycin (1 μg/ml) to the growth medium. Following 10 days of selection, cell colonies were pooled together and cultured in plates containing DMEM, 10% FBS, and puromycin. The protein expression of APE1 was determined by Western blot analysis.

### Immunoblot analysis

Cells were lysed in RIPA buffer (50 mM Tris-HCl buffer, pH 7.4, 150 mM NaCl, 1% Triton X-100, 1% sodium deoxycholate, and 0.1% SDS) supplemented with 1 × Halt protease inhibitor cocktail and 1 × Halt phosphatase inhibitor cocktail (Pierce, Rockford, IL). The Bio-Rad Protein Assay (Bio-Rad, Hercules, CA) was utilized to determine protein concentrations prior to analysis. Proteins were separated by sodium dodecyl sulfate polyacrylamide gel electrophoresis and transferred to Protran nitrocellulose membranes (Whatman, Boston, MA). Membranes were probed with specific primary antibodies (1:1000 dilution) and horseradish peroxidase-conjugated secondary antibodies (Cell Signaling). Protein bands were visualized using Immobilon Western Chemiluminescent HRP Substrate Detection Reagent (Millipore, Billerica, MA). Gel loading was normalized for equal β-actin.

### Immunohistochemistry

Tissue microarrays (TMA) containing 130 de-identified archival cases of EACs as well as normal stomach, normal esophagus, and dysplastic and non-dysplastic BE was constructed by Tissue Pathology Core at Vanderbilt University Medical Center, Nashville, TN. All tissue samples were histologically verified and representative regions were selected for inclusion in the TMA. All of the adenocarcinoma samples were collected from either the GEJ or lower esophagus and ranged from well-differentiated to poorly-differentiated, stages I to IV, with a mix of intestinal- and diffuse-type tumors. All adenocarcinomas were classified according to the recent guidelines of the UICC TNM classification system. The patients' age ranged from 34–84 years (median at 65 years). Tissue cores with a diameter of 0.5 mm were retrieved from the selected regions of the donor blocks and punched to the recipient block using a manual tissue array instrument (Beecher Instruments, Silver Spring, MD). Each tissue sample was represented by three tissue cores on the TMA. Sections (5 μm) were transferred to polylysine-coated slides (SuperFrostPlus, Menzel-Gläser, Braunschweig, Germany). The resulting TMA was used for IHC analysis utilizing rabbit anti-APE1 polyclonal antibody (Cell Signaling). De-waxing and rehydration by descending concentrations of ethanol was followed by antigen retrieval (20 min in a microwave, 450 W, 10 mM EDTA, pH 8.0). Blocking was performed with 10% goat serum for 10 min. The sections were incubated overnight with APE1 primary antibody, followed by washing in PBS and 1 h incubation with anti-rabbit secondary antibody. As a chromogen substrate, the Vectastain ABC-AP KIT (vector; Alexis, Gruenberg, Germany) was utilized, and the specimens were counterstained with hematoxylin. Specificity of immunostaining was checked by replacing the primary antibody with non-immune serum. Tissues with no evidence of staining, or only rare scattered positive cells less than 3%, were recorded as negative. The overall intensity of staining was recorded as that for the core with the strongest intensity. Immunohistochemical results were evaluated for intensity and frequency of staining. The immunoreactivity of the samples tested was assessed by a trained pathologist and scored for intensity and frequency. The intensity of staining was graded as 0 (negative), 1 (weak), 2 (moderate), and 3 (strong). The frequency was graded from 0 to 4 by the percentage of positive cells as follows: grade 0, < 3%; grade 1, 3–25%; grade 2, 25–50%; grade 3, 50–75%; grade 4, more than 75%. For statistical analysis, APE1 protein intensity and frequency were transformed into a composite expression score (CES) utilizing the formula CES = 4 (Intensity – 1) + Frequency [53]. The range of CES was from 0 to 12.

### Chemicals

A bile acid cocktail was prepared with an equimolar mixture of sodium salts of glycocholic acid, taurocholic acid, glycodeoxycholic acid, glycochenodeoxycholic acid, and deoxycholic acid. In all experiments, 200 μM final concentration of the bile acids cocktail (40 μM of each of the above bile acids) was used. This cocktail reflects the mixture of bile acid to which the distal esophagus is ordinarily exposed during gastroesophageal reflux disease, as previously reported [28, 54].

### Cell viability assay

Cell viability was evaluated with the CellTiter-Glo^®^ Luminescent Cell Viability Assay (Promega, Madison, WI). Cells were seeded at 5000 cells/well density in 96-well plate and cultured for 24 h. Cells were then treated with medium containing acidic bile salts cocktail (200 μM, pH 4) for 30 min. After treatment, cells were cultured in complete medium for another 24 h, and then cell viability was evaluated following the manufacturer's protocol. The results were expressed as relative cell viability compared to the vehicle-treated control.

### Immunofluorescence

For the p-H2AX (S139) and 8-OH-dG immunofluorescence assays, cells were treated with acidic bile salts cocktail (200 μM, pH 4) for 30 min. Cells were then cultured in fresh complete media for 1 h post-treatment. The cells were then immediately fixed with cold methanol (−20°C) for 10 min, followed by incubation in 3% BSA in PBS for 45 min to block the non-specific binding sites. Cells were incubated with p-H2AX (S139) (1:500) or 8-OH-dG (1:500) primary antibodies for 1 h at room temperature. Secondary antibodies conjugated with Alexa Fluor 488 were used at 1:1000 dilutions. The slides were counterstained with 4′, 6-diamidino-2-phenylindole (DAPI) and examined under a fluorescence microscope (Nikon, Melville, NY) for analysis.

### Quantitation of apurinic/apyrimidinic (AP) sites

Cells were non-treated or treated with acidic bile salts (200 μM, pH 4, 30 min) and followed by incubation in complete medium for 3 h post-treatment. Genomic DNAs of cells were isolated with DNAzol (Invitrogen) following the manufacturer's protocol. AP Sites Quantitation Kit (OxiSelect^™^; Cell BioLabs, San Diego, CA) was used to quantitate AP sites. The aldehyde reactive probe (ARP) that reacts specifically with an aldehyde group on the open ring form of AP sites (ARP-derived DNA) was detected with Streptavidin-Enzyme Conjugate. The quantity of AP sites was determined using FLUO Star OPTIMA microplate reader (BMG Labtech, Cary, NC) at 450 nm by comparing with a standard curve of predetermined AP sites. All DNA samples were assayed in duplicate.

### Comet assay

DNA single- and double-strand breaks levels were evaluated using a Comet Assay Kit (Trevigen) under alkaline condition following the manufacturer's instructions. Briefly, cells were non-treated or treated with acidic bile salts (200 μM, pH 4) for 30 min; followed by recovery in complete media for 3 h. Cells were suspended at 10^5^ cells/ml in PBS and mixed with Comet Agarose at 1:10 ratio (v/v). The cell mixture (75 μl) was immediately layered onto a comet slide. The slide was maintained at 4°C for 15 min for gelling and then immersed in Lysis Buffer at 4°C for 30 min. The slide was then placed in Alkaline Solution for 30 min in the dark and electrophoresis was performed at 1 V/cm and 300 mA. After the electrophoresis, the slide was rinsed with H_2_O, immersed in 70% ethanol for 5 min and allowed to air dry. The slide was then stained with Vista Green DNA Dye and comet “tails” were visualized with a fluorescence microscope. Extent tail moment values of comet assay were quantitated using the ImageJ software (NIH) from a minimum of 50 individual cells.

### Apoptosis analysis

Cells (10^5^ cells per well) were seeded in triplicate in 6-well plates. The next day cells were non-treated or treated with acidic bile salts (200 μM, pH 4) for 30 min and followed by recovery in complete media for 3 h. Cells were then harvested and stained with Annexin-V and propidium iodide (PI) (BioVision, Mountain View, CA). The cells were washed with PBS and re-suspended in a binding buffer (HEPES buffered saline solution supplemented with 2.5 mM CaCl2), and then subjected to fluorescence-activated cell sorting (FACS) analysis by a flow cytometer (Becton Dickinson). Apoptotic cell death was determined by counting the cells that stained positive for Annexin-V.

### Statistical analysis

The results were expressed as the mean ± SD. The statistical significance of the data was determined by either the parametric unpaired Student's *t* test or two-way ANOVA followed by Bonferroni *post hoc* test. Differences ≤ 0.05 are considered significant.

## SUPPLEMENTARY MATERIAL FIGURES, TABLE AND REFERENCES


