# Supplementary material for: APE1-mediated DNA damage repair provides survival advantage for esophageal adenocarcinoma cells in response to acidic bile salts
Source: Oncotarget. 2016 Feb 25;7(13):16688–702. doi: 10.18632/oncotarget.7696 (PMC4941344; doi:10.18632/oncotarget.7696)
Supplement: Supplementary file 1 [file oncotarget-07-16688-s001.pdf]

APE1-mediated DNA damage repair provides survival advantage for esophageal adenocarcinoma cells in response to acidic bile salts

Supplementary Materials

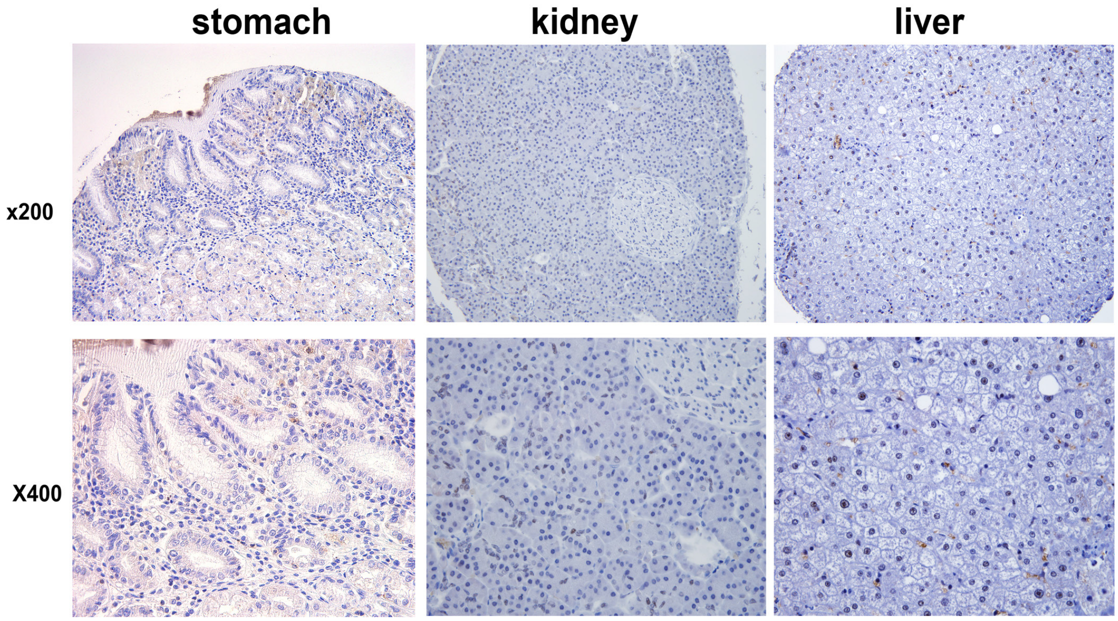

Supplementary Figure S1: Evaluation of APE1 IHC staining in human normal tissues IHC staining for APE1 in normal human stomach, kidney, and liver tissues. Normal stomach and kidney are negative for APE1 immunostaining, whereas normal liver has weak nuclear staining in few cells.

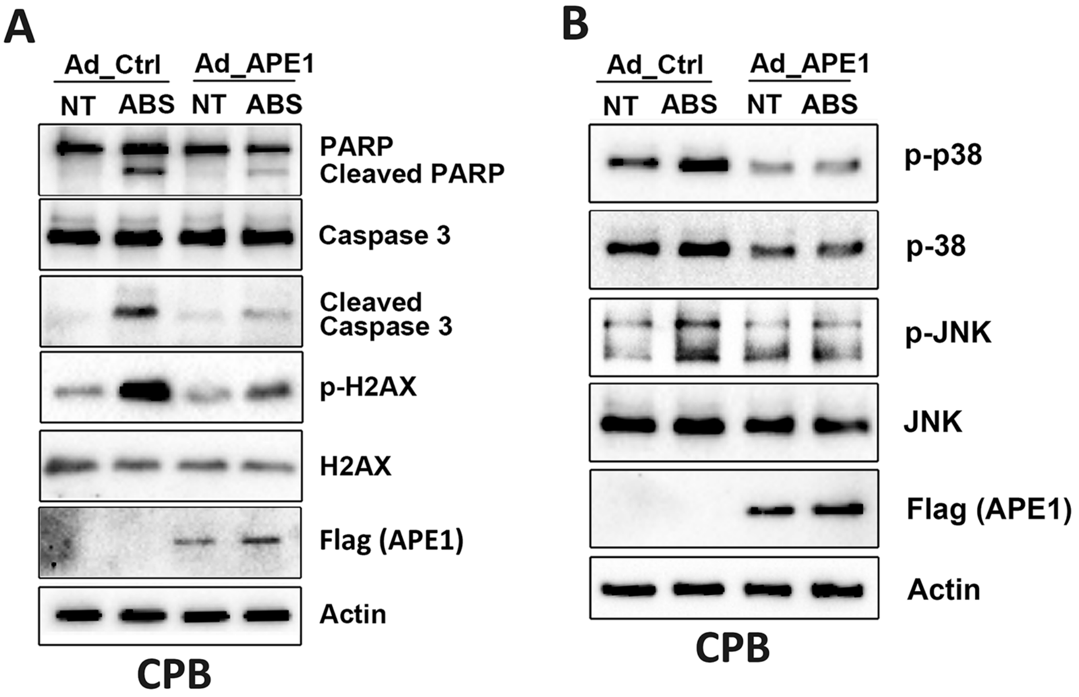

Supplementary Figure S2: APE1 suppresses acidic bile salts-induced DNA damage and apoptosis through regulation JNK and p38 signaling in HGD. CPB cells (HGD) were non-treated or treated with acidic bile salts (200 µM, pH 4) for 30 min, followed by recovery in complete media for 3 h. Cell lysates were subjected to Western blot analyses of the indicated proteins (panels **A** and **B**). These data suggest that APE1 prosurvival function also occurs in HGD cells, similar to EAC cells.

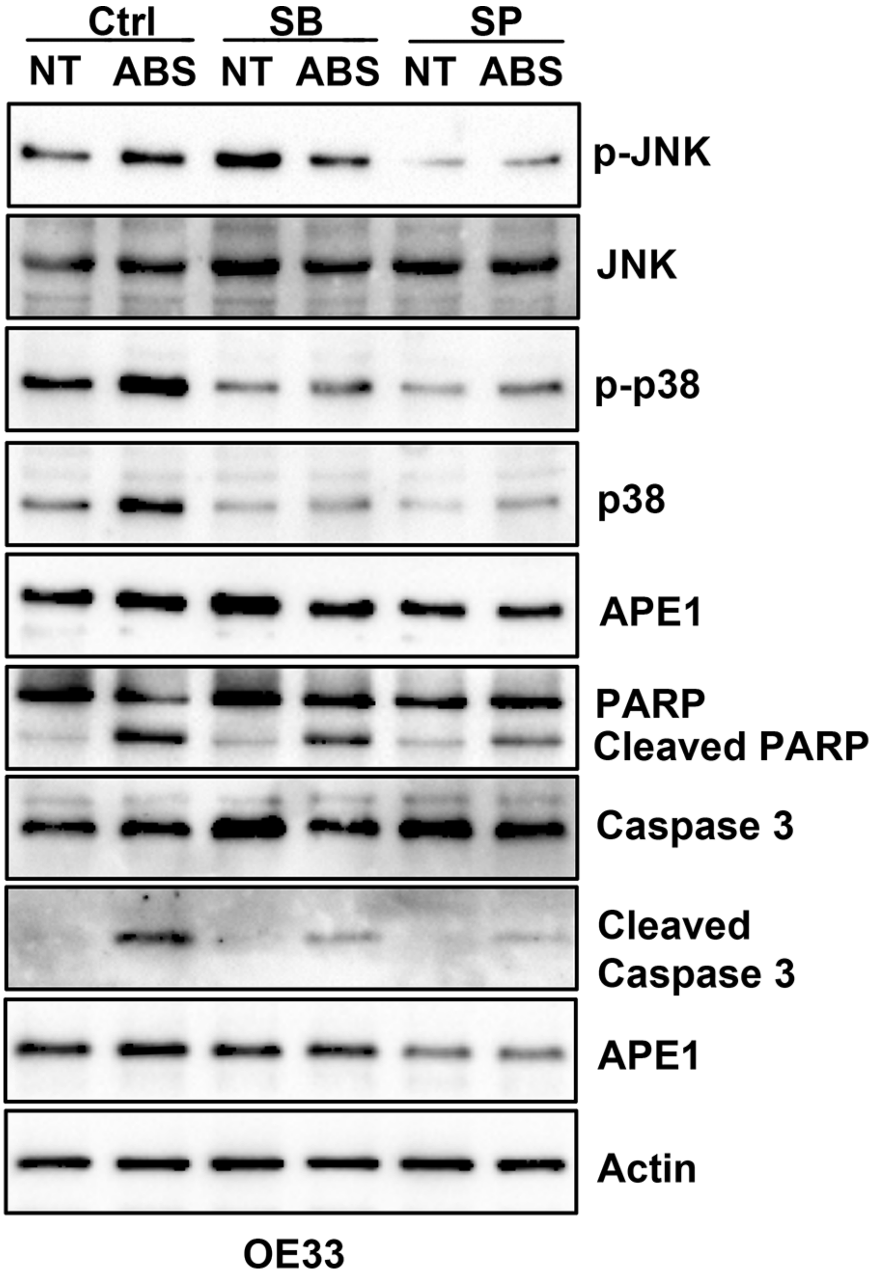

Supplementary Figure S3: Acidic bile salts-induced apoptotic cell death requires activation of JNK and p38 kinases in OE33 cells. OE33 cells were non-treated or treated with acidic bile salts (200 µM, pH 4) alone or in combination with a JNK inhibitor, SP600125 (SP) (2.5 µM) , a p38 inhibitor, SB203580 (SB) (2.5 µM), or vehicle DMSO (V) for 30 min, followed by recovery in complete media for 3 h post-treatments. Cell lysates were then subjected to Western blot analyses of the indicated proteins.

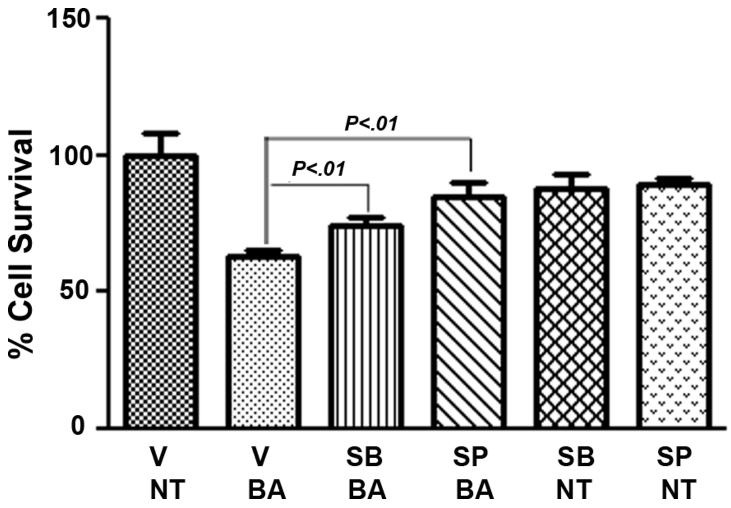

Supplementary Figure S4: Inhibition of JNK or p38 kinases protects against acidic bile salts-induced cell death in EAC cells. OE33 cells were non-treated or treated with acidic bile salts (200 µM, pH 4) alone or in combination with JNK inhibitor SP (2.5 µM), p38 inhibitor SB (2.5 µM) or vehicle DMSO (V) for 30 min, followed by recovery in complete media for 24 h post-treatments. Cell lysates were then subjected to the CellTiter-Glo® Luminescent Cell Viability Assay.

Supplementary Table S1: Immunohistochemistry analysis of APE1 expression levels on esophageal tissue microarrays

	CES Score		
	**0–3**	**4–8**	**9–12**
**NG**	9	1	0
**NE**	17	2	0
**BE**	17	5	0
**BD**	2	7	2
**EAC****	60	36	34

CES [1], composite expression score; NG, normal gastric; NE, normal esophagus; BE, non-dysplastic Barrett's esophagus; BD, Barrett's dysplasia; EAC, esophageal adenocarcinoma. $^{**}P < .01$.

REFERENCES

1. Mukherjee K, Peng D, Brifkani Z, Belkhiri A, Pera M, Koyama T, Koehler EA, Revetta FL, Washington MK, El-Rifai W. Dopamine and cAMP regulated phosphoprotein MW 32 kDa is overexpressed in early stages of gastric tumorigenesis. Surgery. 2010; 148:354–363.
